# Conceptualization of pain in Croatian adults: a cross-sectional and psychometric study

**DOI:** 10.3325/cmj.2024.65.472

**Published:** 2024-12

**Authors:** Snježana Schuster, Iva Lončarić Kelečić, Morana Bilić, Margareta Begić, Joshua W. Pate

**Affiliations:** 1 Department of Physiotherapy, University of Applied Health Sciences Zagreb, Zagreb, Croatia; 2 University Hospital Center Zagreb, Zagreb, Croatia; 3 Department of Health Psychology, University of Applied Health Studies, Zagreb, Croatia; 4 Graduate School of Health, University of Technology Sydney, Sydney, Australia

## Abstract

**Aim:**

To ascertain whether Croatian respondents’ knowledge on pain aligns with modern pain science, and determine the measurement properties of the Croatian version of the Concept of Pain Inventory for Adults (COPI-Adult).

**Methods:**

A cross-sectional, online survey was used to collect the respondents' sociodemographic, clinical, and COPI-Adult (CRO) data (n = 509). A Pearson correlation coefficient test was used to assess the correlations between sociodemographic, clinical, and COPI-Adult (CRO) data. Confirmatory factor analysis and Cronbach's coefficient, based on classical test theory, were used to determine the measurement properties of the questionnaire.

**Results:**

The average COPI-Adult (CRO) score was 35.91 ± 5.8 out of 52 and it was similar in respondents with (36.52 ± 6.01) and without (35.36 ± 5.57) formal medical/health care education. Respondents exhibited a reductionist understanding of pain as a result of structural damage. Higher COPI-Adult scores were very weakly correlated with formal medical/health care education, younger age, lower pain intensity, higher pain knowledge self-assessment, and higher education level. Formal medical education significantly moderately correlated with pain knowledge self-assessment (r = -0.425; *P* < 0.001). One-factor COPI-Adult (CRO) model revealed significant factor loadings of each item (*P* < 0.001) and good internal consistency (Cronbach α = 0.803).

**Conclusions:**

Croatian respondents' concept of pain aligns with their objective knowledge, but only partially with modern pain science. This indicates the need to bridge the gap between traditional and contemporary understandings of pain in the Croatian population. One-factor COPI-Adult (CRO) inventory serves as the first questionnaire for assessing the concept of pain among Croatian adults.

Modern pain science highlights four essential points regarding the biology of pain: (i) pain does not provide a measure of the tissue state; (ii) it is modulated by many factors from across bodily and psychosocial domains; (iii) the relationship between pain and the tissue state becomes less predictable as pain becomes recurrent; and (iv) pain can be conceptualized as a conscious correlate of the implicit perception of tissue danger (1). Consequently, pain should be understood as an actively assembled experience based on multiple information sources, not only nociceptive, reflecting both conscious and non-conscious estimates of body protection needs (2,3). Essentially, pain is a complex experience that cannot be quantified merely by the condition of tissue or injury, but is shaped by various biological, psychological, and social factors (1). Recognizing these contemporary understandings of pain may be crucial for both patients and health professionals. Understanding pain biology changes how people think about pain, reduces its threat value, and improves its management (4-7). Health professionals must be knowledgeable about pain to be able to manage it effectively (8). However, many misconceptions about pain persist in society, and pain remains one of the most misunderstood and undertreated medical problems (9).

Musculoskeletal pain is a prevalent problem across Europe, both in men and women, particularly in people with lower socioeconomic status (10). Patients with the most common underlying disorders, such as low back pain (LBP), as well as their health care providers (11,12), frequently understand pain as being a direct consequence of physiological dysfunction (11,13-16) and a “marker of tissue damage“ (2), contrary to understandings advocated by modern pain science (17). Pain understanding is a complex cognitive process in which the individual's knowledge and beliefs play essential roles; however, beliefs about pain, often held as steadfast convictions, may not always be rational and could persist despite facts (18). Under the umbrella of pain science and conceptual change theory, knowledge and beliefs are essential for pain conceptualization (1,19). Pain neuroscience education integrates pain science and conceptual change theory and challenges traditional views, emphasizing that pain is not a straightforward reflection of tissue damage but rather an output of the brain, shaped by multiple central and peripheral processes and reflecting a perception of threat (2,4,17). Since understanding pain involves complex cognitive processes heavily influenced by knowledge and beliefs, pain science education seeks to align these understandings with scientific insights, empowering patients to reframe their pain and adopt more effective coping strategies (2).

With an aim to evaluate the effectiveness of pain science education for pain conceptualiaztion, Pate et al (19) developed The Concept of Pain Inventory for Adults (COPI-Adult), a brief questionnaire identifying misconceptions regarding pain. The authors revealed valuable findings about adults' knowledge alignment with modern pain science and concept-related factors. The value of COPI-Adult has been recognized on the European scene of pain science; a Danish-validated version exists (20), and other versions are expected.

As there has been no study exploring the concept of pain in Croatian adults, this study aimed to explore the alignment of Croatians' knowledge with modern pain science and to identify sociodemographic and clinical factors related to the concept. Additionally, it tested the measurement properties of the Croatian (CRO) COPI-Adult.

## RESPONDENTS AND METHODS

This cross-sectional, psychometric study was conducted online from July until August 2023. It was approved by the Ethics Committee of the University of Applied Health Sciences, Zagreb, and conforms to the principles established by Croatian and European regulations (21), including the Declaration of Helsinki (22).

The survey, available on Google Forms, was disseminated through social networks. The researchers initiated the sharing of the survey information. Respondents were recruited via snowball sampling and completed a self-report questionnaire consisting of sociodemographic and clinical questions, and a concept of pain inventory. When submitting responses, respondents also provided informed consent. The target group were Croatian residents, >18 years old, native Croatian speakers, cognitively and physically able to answer all the questionnaire requirements, and with internet access. The exclusion criterion was ambiguous data.

We aimed to obtain a convenient and diverse sample of Croatians. A minimum sample size of 260 was established based on sample size recommendations for factor analysis and the suggested minimum samples ranging from 3 to 20 times the number of inventory items (23).

### Research instruments

The survey gathered demographic data on age (number of years at the time of the study), sex (male/female), education level (elementary school, high school, college, university), and formal medical/health care education (yes/no). It also gathered clinical data on chronic or recurring pain (yes/no), its location (ie, neck, back, etc), duration (categories of duration), and current intensity (ordinal scale). The survey also asked about previous pain science education, with respondents providing information on formal education in medicine/health care, as well as the level of their professional education (high school, college, or university). Sociodemographic and clinical questions were close-ended, and the respondents had to select one of the answers. Age information was self-reported. If the respondents did not want to answer specific questions, they were instructed to leave them blank.

Current pain intensity in respondents with chronic or recurrent pain was assessed with the Numeric Pain Rating Scale. This unidimensional 11-point measure of pain intensity ranges from 0 (no pain) to 10 (worst pain imaginable), with the score 1–3 indicating mild, 4–6 indicating moderate, and >7 indicating severe pain (24,25).

Knowledge and beliefs regarding pain were assessed with the 13-item COPI-Adult (CRO). Items were rated on a 5-point Likert scale (0 = strongly disagree, 1 = disagree, 2 = unsure, 3 = agree, 4 = strongly agree) (19). Sum scores ranged from 0-52; higher COPI-Adult scores reflect greater alignment of knowledge and beliefs with contemporary pain science, with no reversed scores. The originators of COPI-Adult considered it suitable for persons with and, particularly, without a pain science education. The inventory was used with permission and in communication with its principal originator (JP). The Croatian version of COPI-Adult was translated and completed in February 2023. The original questionnaire was translated from English to Croatian by a professional translator, and the final version was further reviewed by a team consisting of an associate professor expert in pain science, a physiotherapist and a psychologist, PhD candidates researching pain science, one PhD student in physiotherapy, and one peer patient without formal medical/health care education. After a thorough review, the COPI-Adult (CRO) was deemed as adequately translated and contextually adapted; hence, it was approved for further research.

Similar to the self-assessment of knowledge in the Evidence-Based Practice Profile questionnaire (26), we assessed participants' perceived familiarity with contemporary pain science by asking them to rate their knowledge about pain and related factors on a scale from 1 (no knowledge) to 5 (excellent knowledge).

### Statistical analysis

Statistical analysis was conducted with SPSS (version 25), SPSS AMOS (both IBM Corp., Armonk, NY, USA), and JASP (version 0.18.2.0). Regarding the original COPI-Adult inventory (19), methodologies compatible with evaluating the measurement properties of the reflective model (27) were applied. COSMIN (27) and STROBE guidelines (28) were followed.

Descriptive statistics were used to assess the distribution of responses. Categorical variables are presented as numbers and percentages, and continuous variables as means and standard deviations (SD). For COPI-Adult (CRO) scores, skewness, kurtosis, corrected item correlation, and factor loadings were determined. To assess skewness, established cutoffs were used: values between -0.5 and 0.5 suggested almost symmetrical data distribution, values between -1 and -0.5 suggested negative skewness, and those between 0.5 and 1 suggested positive skewness. Skewness lower than -1 (negatively skewed) or greater than 1 (positively skewed) implies highly skewed data (29). To assess correlations between COPI-Adult (CRO) variables and sociodemographic and clinical variables, the Pearson correlation coefficient test was used at a two-tailed 0.01 and 0.05 significance level. Correlation coefficients were interpreted in the following way: <0.20 – very weak, 0.20-0.39 – weak, 0.40-0.59 – moderate, 0.60-0.79 – strong, and >0.80 – very strong (30).

Measurement properties of the test were evaluated based on classical test theory (CTT) (31) as proposed for the reflective model of the COPI-Adult inventory. Confirmatory factor analysis (CFA) was conducted to assess structural validity and determine whether the questionnaire retained the original, author-proposed one-factor model of the COPI-Adult (19). The χ^2^ was calculated as an indicator of model fit. The ratio of the χ^2^ value and the number of degrees of freedom lower than 5 indicated a good fit for the model. The maximum likelihood method with robust standard errors was used to estimate the parameters of the confirmatory models: comparative fit index (CFI), Bentler-Bonett normed fit index (NFI), Tucker-Lewis index (TLI), root mean square error of approximation (RMSEA), English goodness of fit (GFI), and English standardized root mean square (SRMR), as absolute and relative model fit indicators. The model was considered as good if CFI, NFI, and TLI values were equal to or greater than 0.90; GFI equal to or greater than 0.85; and SRMR and RMSEA values ranged from 0.05 to 0.10 (32). In case of unsatisfactory model fit, modification indices (MI) were computed to provide more information about the model. If theoretically justifiable, error covariance was added over item pairs with high MI values to improve model fit (33). Internal consistency was checked by dimension homogeneity testing and was expressed by Cronbach's coefficient α. Commonly, α values in the range of 0.6-0.7 are acceptable, while the values of 0.8 or higher are considered good or very good (34). Coefficients were complemented by adjusted item-total correlations (ITCs). An ITC>0.30 indicated acceptable internal consistency, while an ITC below <0.30 was considered unacceptable (19,35).

## RESULTS

The study enrolled 509 respondents, with all the responses included. In the overall sample, 224 participants (44%) had formal medical/health care education. The mean age of the entire sample was 41.76 ± 12.09 years. Most of the respondents were women (80.6%), had university-level education (54.8%), and reported chronic or recurrent pain (61%). A total of 15.1% reported pain lasting longer than 10 years, and 28.3% reported pain lasting up to 10 years. The most prevalent pain type reported across both groups was lower back pain (LBP) at 37.5%, followed by shoulder and neck pain (13.9%).

The group without formal medical/health care education comprised 285 respondents (mean age: 42.59 ± 12.14 years). The majority were women (79.2%), had a university education (53.6%), experienced chronic or recurrent pain (61.0%), and suffered from LBP (60.2%). A total of 28.1% reported pain lasting from 3 to 10 years, while 28.8% experienced pain lasting more than 10 years. The mean pain intensity was 4.28 ± 2.27, which indicates moderate pain levels. The average self-assessed pain knowledge score was 3.16 ± 1.15.

The group with formal medical/health care education comprised 224 respondents (mean age: 40.71 ± 11.96 years). The majority were women (82.1%), held a university degree (57.1%), reported chronic or recurrent pain (60.7%), and suffered from LBP (51.9%). A total of 31.1% reported pain lasting over 3 to 10 years, while 23.5% experienced pain lasting more than 10 years. The mean pain intensity was 3.96 ± 2.21, and the mean self-assessment pain score was 4.17 ± 0.92.

The group with no formal education had a mean COPI-Adult (CRO) score of 35.36 ± 5.57, while the group with formal education had a mean score of 36.52 ± 6.01. The difference between groups was not significant (Table 1, Table 2).

**Table 1:** Characteristics of respondents stratified by formal education

	No. (%) of patients with	
Variable	no formal medical/health care education (N = 285)	formal medical/health care education (N = 224)
**Sex**		
female	226 (79.2)	184 (82.1)
male	59 (20.7)	40 (17.9)
total	285 (100)	224 (100)
**Highest level of education**		
elementary	3 (1.0)	-
high-school	92 (32.2)	38 (17.0)
college	37 (12.9)	58 (25.9)
university	153 (53.6)	128 (57.1)
total	285 (100)	224 (100)
**Chronic or recurrent pain**		
yes	174 (61.0)	136 (60.7)
no	111 (38.9)	88 (39.3)
total	285 (100)	224 (100)
**Pain location** (N = 166/133)^†^		
back	100 (60.2)	69 (51.9)
shoulder/neck	27 (16.3)	35 (26.3)
leg/foot	14 (8.4)	12 (9.0)
arm/hand	3 (1.8)	5 (3.8)
head/face/jaw	10 (6.0)	7 (5.3)
abdomen	6 (3.6)	-
other	6 (3.6)	5 (3.8)
total	166 (100)	133 (100)
**Pain duration** (N = 160/132)^†^		
0 to 3 months	16 (10.0)	15 (11.4)
3 to 6 months	13 (8.1)	9 (6.8)
6 to 12 months	6 (3.8)	12 (9.1)
1 to 3 years	34 (21.2)	24 (18.2)
3 to 10 years	45 (28.1)	41 (31.1)
>10 years	46 (28.8)	31 (23.5)
Total	160 (100)	132 (100)
Continuous variables	Mean (standard deviation)	Mean (standard deviation)
Age (years)	42.59 (12.14)	40.71 (11.96)
Current pain intensity (0-10)*	4.28 (2.27)	3.96 (2.21)
Pain Knowledge Self-Assessment (1-5)*	3.16 (1.15)	4.17 (0.92)
COPI-Adult Score (0-52)*	35.36 (5.57)	36.52 (6.01)

*A higher score implies higher pain intensity, knowledge and COPI-Adult score. Percentages may not add up to 100% because of rounding.

^†^Percentages are based on the subset of respondents who reported pain and provided valid data for these variables (n = 166 for no formal education; n = 133 for formal education; n = 160 and n = 132 for pain duration, respectively). Percentages for other categorical variables are calculated based on the total group size.

**Table 2:** The reported source of received pain education within formal medical/health care education (N = 224)

Degree in medical/health care profession, education level achieved	N (%)
Yes, university	98 (19.3)
Yes, high school	42 (8.3)
Yes, college	84 (16.5)
No	285 (56)
Total	509 (100)

The item score distribution was approximately normal in all aspects. Kaiser-Meyer-Olkin’s measure of sampling adequacy was 0.84 (Table 3).

**Table 3:** The Concept of Pain Inventory (COPI) for Adults (CRO) item score distribution

**Item**	**Mean**	**Standard deviation**	**Skewness**	**Kurtosis**	**Corrected item-total correlations**	**Factor loading**
**1**	**2.94**	**0.83**	**-0.82**	**0.95**	**0.47**	**0.416**
**2**	**3.02**	**0.75**	**-0.88**	**1.47**	**0.55**	**0.437**
**3**	**2.72**	**0.81**	**-0.76**	**0.91**	**0.33**	**0.330**
**4**	**2.55**	**0.96**	**-0.68**	**0.11**	**0.41**	**0.430**
**5**	**2.46**	**0.85**	**-0.49**	**0.28**	**0.40**	**0.396**
**6**	**2.65**	**0.89**	**-0.65**	**0.13**	**0.36**	**0.335**
**7**	**3.15**	**0.63**	**-0.46**	**1.03**	**0.51**	**0.377**
**8**	**2.8**	**0.73**	**-0.53**	**0.51**	**0.45**	**0.351**
**9**	**2.46**	**0.86**	**-0.38**	**-0.08**	**0.40**	**0.380**
**10**	**2.8**	**0.77**	**-0.58**	**0.47**	**0.49**	**0.436**
**11**	**2.81**	**0.81**	**-0.45**	**-0.03**	**0.415**	**0.374**
**12**	**2.62**	**0.89**	**-0.68**	**0.25**	**0.40**	**0.431**
**13**	**2.81**	**0.79**	**-0.76**	**1.03**	**0.49**	**0.433**
**COPI total**	**35.91**	**5.8**	**0.14**	**0.49**	**-**	**-**


Higher COPI-Adult scores very weakly correlated with formal medical/health care education (r = -0.106, *P* < 0.05), older age (r = -0.146, *P* < 0.001), lower current pain intensity (r = -0.168, *P* < 0.001), higher self-assessed pain knowledge (r = 0.133, *P* < 0.001), and higher overall education levels (r = 0.147, *P* < 0.001). Notably, formal medical education demonstrated a significant moderate negative correlation with pain knowledge self-assessment (r = -0.425, *P* < 0.001). There were no significant correlations between COPI-Adult scores and chronic pain status (r = 0.031) or sex (r = 0.038) (Table 4).

**Table 4:** Correlations between the Concept of Pain Inventory (COPI) for Adults (CRO), sociodemographic, and clinical variables

	1	2	3	4	5	6	7	8
**1. Formal medical education (No)**	1	0.078	0.071	-0.425^†^	-0.036	0.013	-0.188^†^	-0.106*
**2. Older age (per year)**		1	0.121*	0.032	0.115^†^	-0.293^†^	-0.023	-0.146^†^
**3. Current pain intensity (0-10)^‡^**			1	0.086	0.072	-0.092	-0.197^†^	-0.168^†^
**4. Pain knowledge self-assessment (1-5)**				1	0.047	-0.109*	0.060	0.133^†^
**5. Sex (male)**					1	-0.096*	0.075	0.038
**6. Chronic pain (yes)**						1	0.083	0.031
**7. Highest education level^§^**							1	0.147^†^
**8. COPI Adult TOTAL**								1

*Correlation is significant at the 0.001 level (two-tailed)

†Correlation is significant at the 0.005 level (two-tailed).

‡Higher scores indicate higher levels of pain intensity.

§Education level was treated as a continuous variable, scored from 1 to 4.

**Figure 1:**
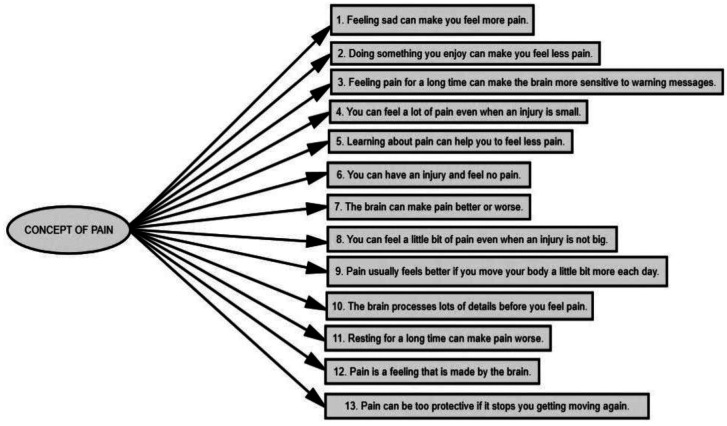
Confirmatory factor analysis model using Concept of Pain Inventory (COPI) for Adults (CRO).

**Figure 2:**
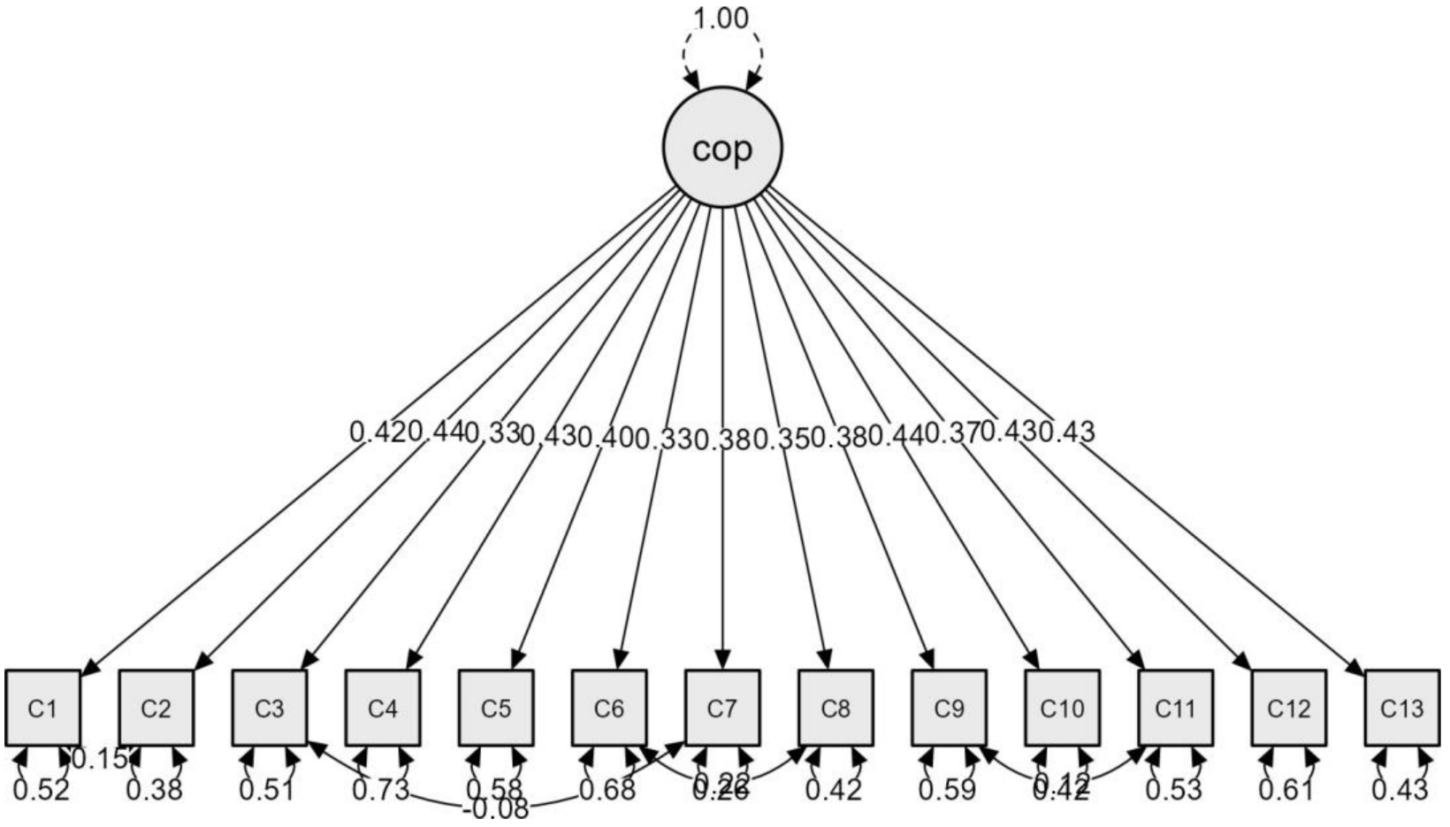
Concept of Pain Inventory (COPI) for Adults (CRO) confirmatory factor analysis model plot with standardized factor loadings and associated covariances between items.

The COPI-Adult (CRO) showed “good” internal consistency, with Cronbach α = 0.03. All the corrected ITCs were >0.3. CFA (Figure 1, Figure 2, Table 5) revealed an acceptable fit of the tested model, confirming that, COPI-Adult (CRO) retained the original one-factor structure. Standardized factor loadings of each item (Figure 2) were significant (*P* < 0.01) in the model, verifying the “concept of pain” as a latent variable underlying all items in the COPI-Adult (CRO). MI suggested covariances for several item pairs. Covariances were related to items 1 and 2 (0.15), items 3 and 7 (-0.08), items 6 and 8 (0.26), and items 9 and 11 (0.12). The criteria for a good model (Table 5) were acceptable, with all values in the desirable range, except TLI and NFI, which were slightly below the acceptable limit.

**Table 5:** Model fit measures derived from confirmatory factor analysis of potential factor structures for the Concept of Pain Inventory (COPI) for Adults (CRO)

Model	χ2	df^†^	χ^2^/df	Comparative fit index	English Goodness of fit	Tucker-Lewis index	Bentler-Bonett normed fit index	Root mean square error of approximation	Standardized root mean square
One factor	187.652*	61	3.07	0.90	0.99	0.87	0.86	0.06	0.05

*P < 0.001.

^†^degrees of freedom.

## DISCUSSION

In this study, the concept of pain aligned with respondents’ objective knowledge, albeit only partially with modern pain science. The COPI Adult (CRO) score was weakly associated with PSE education, knowledge self-assessment, age, and current pain intensity. This is the first study in Croatia and one of the few in Europe that explored the concept of pain in adults.

The survey, disseminated mainly via Facebook, had a broad reach with over five hundred responses. Most respondents were well-educated women of a younger mature age, both with and without formal medical/health care education. About two-thirds of the respondents reported chronic or recurring LBP of moderate intensity (24,25) for up to ten years. LBP is the main overall health burden contributor, responsible for 7.4% of global years lived with disability (DALYs) (36). In 2019, it was among the ten most common causes of disability in Croatia and ranked second in terms of DALYs (37).

The knowledge on pain of Croatian respondents, regardless of their formal education, partially aligned with modern pain science. The mean score in our respondents with (36.52 ± 6.01) and without (35.36 ± 5.57) PSE was similar to that in English-speaking respondents without PSE (35.9 ± 5.2) but lower than in English-speaking respondents with PSE (41.1 ± 6.8) (19). In their study, Pate et al focused specifically on identifying the sources of PSE, providing a clear definition for participants to ensure consistency in understanding what constituted PSE (19). In contrast, our research, while also centered on prior PSE, focused more on formal health care education and the educational levels of respondents without providing an explicit definition of PSE.

The COPI-Adult score of Croatian respondents with completed medical/health care education was significantly lower than that of Australian health care students (median 39; IQR 36–44) (38). The respondents in our study most frequently had misconceptions about the relationship between the intensity of pain and the size of injury and the effect of education and movement on pain sensation. In other words, they believed that pain was a result of a larger structural damage, which cannot be treated other than by rest. Our study found higher COPI-Adult scores to be weakly correlated with formal medical/health care education, younger age, lower current pain intensity, higher pain knowledge self-assessment, and higher education level, while the correlation between formal medical/health care education and pain knowledge self-assessment was moderate and significant. These findings suggest that participants with formal medical/health care education tended to assess their pain knowledge higher than those without it. Pate et al (19) showed that higher COPI-Adult scores were associated with pain interference, female sex, the highest level of education, and current pain intensity; however, only in respondents with PSE.

Although our findings, like those by Pate et al (19), imply a relationship between PSE and higher results on the COPI-Adult, Croatian respondents, particularly those with formal medical/health care education, still had insufficient pain knowledge. This issue is important from the perspective of health care quality and patient outcomes. Healthcare professionals who have misunderstandings about pain are more likely to recommend treatments that are not supported by evidence, while policymakers who harbor such misunderstandings may allocate resources to evidence-unsupported pathways (39). This might create a chain reaction perpetuating low-quality care, resulting in poorer outcomes for all health care stakeholders (39). Health professionals without adequate knowledge about pain (8) may provide conflicting information to their patients, which may serve as an iatrogenic pain contributor (40). Our respondents’ pain knowledge self-assessment had a low agreement with their COPI-Adult (CRO) score, which implies that respondents with formal medical education overestimate their knowledge. It also confirms that self-ratings can be higher than objectively assessed knowledge (41). This finding may motivate health care providers to acknowledge their limitations and seek additional education or consultation when required.

The COPI-Adult inventory was evaluated in its original (19) and Danish (20) versions. Considering the assumed significant response at a single time point and the risk of non-response, we *a priori* decided to evaluate the measurement properties of COPI-Adult (CRO) on cross-sectional data and using CTT. CFA revealed acceptable fit of the tested model and significant standardized factor loadings for each item. It verified the “concept of pain” as the latent variable underlying all items in the inventory, thus confirming the one-factor structure of the COPI-Adult (CRO). In the original COPI-Adult, one-factor structure was also confirmed by CFA, with acceptable internal consistency (38). However, although our criteria for a good model were acceptable, TLI and NFI were slightly below the standard limit. This may be explained by the sample sizes for CFA and MI, which suggested covariances for several item pairs. As seen in the COPI-Adult (CRO), items 1 and 2 relate to the influences of emotions on pain (19); items 3 and 7 relate to the brain and pain; items 6 and 8 relate to injury and pain; and items 9 and 11 relate to movement and rest. Since each item pair addresses the same pain-related dimension, modifying the model by adding residual covariance was theoretically justifiable. The COPI-Adult (CRO) showed good internal consistency with a good Cronbach coefficient and all the corrected ITCs above the agreed benchmark; hence, it was confirmed as acceptable for assessing the concept of pain in Croatian respondents.

The study is subject to several limitations. Although most studies that use survey data rely on CTT, this approach has several theoretical drawbacks that could reduce the generalizability of the findings (42). Nevertheless, the reliability parameters, discrimination, location, or factor loadings are based on a large cohort of Croatian respondents. Our sample is not only large but also comprises native Croatian speakers, which justifies the internal consistency of COPI-Adult (CRO). In contrast, the study by Pate et al (19) included respondents with sufficient English knowledge to complete the survey. Given the nonexistence of a similar inventory in the Croatian language, we could not ascertain convergent and divergent validity.

As almost two-thirds of the participants reported chronic or recurrent pain, a larger sample of healthy individuals is necessary to determine the relevance of the COPI-Adult for this group. Future research on pain conceptualization should explore changes in the COPI-Adult (CRO) in a specific cohort of individuals undergoing contemporary PSE. Furthermore, it should investigate whether individuals with chronic primary musculoskeletal pain (43) or affective disorders exhibit distinct patterns of pain concepts compared with the general population and whether the COPI-Adult can predict patient outcomes. In addition, data should be obtained from multiple time points to ensure the reliability and sensitivity to change of the COPI-Adult (CRO).

In conclusion, the one-factor COPI-Adult (CRO) demonstrated good internal consistency, which makes it the first questionnaire for testing the concept of pain in Croatian adults. This study highlights the need to bridge the gap between traditional and contemporary understandings of pain. Targeted PSE based on identified knowledge gaps and misconceptions can be implemented (38) for students, health professionals (44) and patients, both in clinical practice and in communities through public health campaigns (39).
